# Low Stroke Volume Index in Healthy Young Men Is Associated with the Incidence of Acute Mountain Sickness after an Ascent by Airplane: A Case-Control Study

**DOI:** 10.1155/2020/6028747

**Published:** 2020-11-10

**Authors:** Jingbin Ke, Chuan Liu, Shiyong Yu, Shizhu Bian, Chen Zhang, Jie Yang, Jihang Zhang, Jun Jin, Rongsheng Rao, Ying Zeng, Lan Huang

**Affiliations:** ^1^Institute of Cardiovascular Diseases of PLA, Xinqiao Hospital, Army Medical University, Chongqing 400037, China; ^2^Department of Cardiology, Xinqiao Hospital, Army Medical University, Chongqing 400037, China; ^3^Department of Medical Ultrasonics, Xinqiao Hospital, Army Medical University, Chongqing 400037, China

## Abstract

**Background:**

The aims of this study were to explore the characteristics of left ventricular (LV) functional changes in subjects with or without acute mountain sickness (AMS) and their associations with AMS incidence.

**Methods:**

A total of 589 healthy men were enrolled and took a trip from Chengdu (500 m, above sea level (asl)) to Lhasa (3700 m, asl) by airplane. Basic characteristics, physiological data, and echocardiographic parameters were collected both at Chengdu and Lhasa, respectively. AMS was identified by the Lake Louise Questionnaire Score.

**Results:**

The oxygen saturation (SpO_2_), end-systolic volume index, end-diastolic volume index (EDVi), stroke volume index (SVi), E-wave velocity, and E/A ratio were decreased, whereas the heart rate (HR), ejection fraction, cardiac index (CI), and A-wave velocity were increased at the third day after arrival, as evaluated by an oximeter and echocardiography. However, AMS patients showed higher HR and lower EDVi, SVi, CI, E-wave velocity, and E/A ratio than AMS-free subjects. Among them, SVi, which is mainly correlated with the changes of EDVi and altered LV filling pattern, was the most valuable factor associated with AMS incidence following receiver-operator characteristic curves and linear and Poisson regression. Compared with subjects in the highest SVi tertile, subjects in the middle SVi tertile showed higher multivariable Incidence Rate Ratios (IRR) for AMS with higher incidences of mild headache and gastrointestinal symptoms, whereas subjects in the lowest SVi tertile showed even higher multivariable IRR with higher incidences of all the symptoms.

**Conclusions:**

This relatively large-scale case-control study revealed that the reduction of SVi correlated with the altered LV filling pattern was associated with the incidence and clinical severity of AMS.

## 1. Introduction

High-altitude (HA) exposure has been widely recognized as a source of cardiovascular stress. It has been well characterized that chronic or long-term HA exposure may lead to severe hypoxemia, hypoxic pulmonary hypertension, right ventricular hypertrophy, and/or right heart failure [1], whereas acute or short-term HA exposure may result in a series of cardiovascular adaptive responses, including increases in the heart rate (HR) both at rest and during exercise, progressive elevations of blood pressures in both healthy individuals and hypertensive patients [2, 3], transient pulmonary vasoconstriction, and incremental changes in cardiac contractility and cardiac output [4]. Both the acute adaptation and the chronic remodelling of the cardiac structure and function may be required for facilitating O_2_ delivery in HA travellers and highlanders [5]. In addition, acute HA or hypoxia exposure induces alterations in electrophysiological parameters including decreases in the amplitude of the P/QRS/T waves during a hypoxia exercise test [6] and tachyarrhythmias at HA above 4100 m [7]. However, the characteristics of these cardiovascular changes in patients with acute mountain sickness (AMS) have not been thoroughly illuminated.

The number of sea level residents engaged in HA travel for leisure, sport, or work is progressively increasing. Within the first few days after arriving at HA, AMS, characterized by the presence of headache as well as dizziness, gastrointestinal symptoms, and fatigue, may occur in more than 25% of individuals ascending to 3500 m and in more than 50% of those reaching an altitude above 6000 m [8]. In some individuals, severe or even fatal illnesses may occur, such as high-altitude cerebral edema (HACE), high-altitude pulmonary edema (HAPE), or sudden death [9]. Annually, AMS threatens the well-being and/or life of millions of sojourners and workers at HA [10]. To develop effective strategies for prediction, prevention, and treatment of AMS, persistent efforts have been made for many decades to explore the underlying factors associated with AMS incidence. Until now, established risk factors include the rate of ascent, altitude reached, age, sex, obesity, and individual predisposition [11]. Moreover, it has been reported that high desaturation and low ventilatory response to hypoxia at exercise independently contribute to the development of severe AMS [12]. Although it has been proved that acute HA exposure-induced changes in blood pressures and electrophysiological parameters are not associated with AMS incidence [2, 6], the associations of cardiac function with AMS are still unclear.

Based on these considerations, this relatively large-scale case-control study is conducted to determine the characteristics of left ventricular (LV) functional responses to acute HA exposure and their associations with the incidence of AMS.

## 2. Methods

### 2.1. Participants and Study Procedures

**Figure 1:**
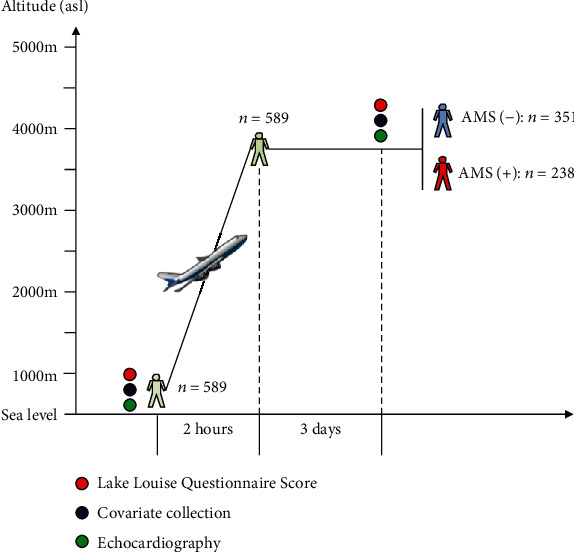
Outline of this study. For the description of the study design, sample size, measurements, and study time points, please refer to the text.

This study was registered with the Center of Chinese Clinical Trial Registration (No.: ChiCTR-RCS-12002232) to determine the effects of acute HA exposure on cardiac functions and their associations with AMS on the Qinghai-Tibet plateau. This study was conducted under an unfavourable condition for field study at HA and a narrow time window between the onset of AMS and HA acclimatization for data collections. However, the inclusion of homogeneous military personnel made the study more feasible. Accordingly, 589 healthy young men aged from 17 to 43 years were included. All the participants who were born or permanently lived in the lowlands (<500 m above sea level (asl)) travelled to Lhasa (Tibet, approximately 3700 m asl) from Chengdu (Sichuan, approximately 500 m asl) by airplane for approximately 2 hours. Data were collected at two time points: within one week before the trip and the third day after arrival (Figure 1). Exclusion criteria were known cardiovascular diseases, any chronic cardiovascular therapy, pulmonary diseases, haematologic diseases, malignant tumours, liver or kidney dysfunction, exposures to altitudes 2500 m asl in the past 6 months, usage of drugs for preventing AMS, history of AMS, history of angioedema, and psychiatric disorders that would interfere with completion of the data collection. Subjects with HACE or HAPE who needed emergent medical interventions were also excluded. All procedures and protocols were approved by the Clinical Research Ethics Board at the Third Military Medical University (Army Medical University) (No.: 2012014) and conformed to the standards set by the Declaration of Helsinki. All subjects volunteered to participate in this study and provided written consent for inclusion.

### 2.2. Identification of AMS Patients

Incidence and severity of AMS were evaluated using the Lake Louise Questionnaire Score (LLQS) according to the newly revised diagnostic standard [13]. The following categories of AMS symptoms were assessed: (1) headache, (2) dizziness/lightheadedness, (3) gastrointestinal symptoms, and (4) fatigue and/or weakness. Participants were asked to complete the self-reported LLQS before sleeping and after awakening, and the highest scores during the stay at HA were considered as the final LLQS. The severity of each AMS symptom was from 0 (no discomfort) to 3 (severe discomfort). Subjects with LLQS ≥ 3 points in the presence of headache were diagnosed as AMS.

### 2.3. Echocardiographic Assessments

Echocardiography examination was performed by registered sonographers by using CX50 ultrasound systems (Philips Ultrasound System, Andover, MA, USA) as previously described [14]. All echocardiographic images were acquired at end-expiration with the subjects in the left lateral decubitus position after at least 10 min of rest to allow them to reach a steady state. Echocardiographic data were recorded on DVDs, and the best images from each study were selected and digitized on a digital image analysis system (Nova Microsonics, Allendale, New Jersey, USA) by two independent investigators who were unaware of the subject's identity and study site.

Two-dimensional guided M-mode measurements of LV end-diastolic and end-systolic volumes (EDV and ESV, respectively) were obtained according to the recommendations of the American Society of Echocardiography [15], which were used for the calculation of the stroke volume (SV) (SV = EDV‐ESV). Cardiac output (CO) was calculated by SV × HR. ESV, EDV, SV, and CO were indexed to the body surface area (BSA, 0.0061 × height(cm) + 0.0128 × weight(kg)‐0.1529), and the results were displayed as ESVi, EDVi, SVi, and CI, respectively. LV ejection fraction (LVEF) was determined in the apical 4- and 2-chamber views using the biplane Simpson method. In the Doppler examinations, the transmitral Doppler LV filling pattern was evaluated from the apical 4-chamber view. The early (E) and late (A) diastolic wave peak velocities and the E/A ratio were determined.

### 2.4. Covariate Collection

Basic information (age, height, body weight, smoking, and drinking status) was recorded. Body mass index (BMI) was calculated as weight (kg)/[height (m)]^2^. Systolic blood pressure (SBP), diastolic blood pressure (DBP), and HR were the mean of two measurements detected by Omron HEM-6200 (Japan) with the subject resting in the sitting position for at least 10 min. The mean arterial pressure (MAP) was calculated by the following formula: MAP = (SBP + 2 × DBP)/3. Oxygen saturation (SpO_2_) was detected with warmed hands at the fingertip with a pulse oximeter (Nonin ONYX OR9500, USA) after at least 10 min of rest and an average of three consecutive measurements with at least 30 s of signal stabilization.

### 2.5. Statistical Analysis

Continuous variables were presented as the mean ± standarddeviation or medians (25th–75th percentile) based on their normality following the Kolmogorov-Smirnov test. Categorical variables were expressed as count and percentage. Analysis of variance or Kruskal-Wallis test was used for a cross-group comparison of continuous variables, and the *χ*^2^ test for independence/Fisher's exact test was used for categorical variables, as appropriate.

Receiver-operator characteristic (ROC) analysis was used to evaluate the ability of potential variables to discriminate AMS incidence by estimating and comparing the area under the curve (AUC) with a corresponding 95% confidence interval (CI). According to the tertiles of SVi among the 589 participants at HA, the SVi was divided into 3 categories: the highest tertile, SVi > 41.45 ml/m^2^; a middle tertile, SVi = 32.66-41.45 ml/m^2^; and the lowest tertile, SVi < 32.66 ml/m^2^. Pearson correlation analysis was used to explore the correlations between continuous variables. Linear regression models were performed to evaluate the significance of any correlations or regression coefficient comparisons. A Poisson model was performed to calculate the Incidence Rate Ratios (IRR) with 95% confidence intervals (CI) for the SVi (continuous and categorical variables) associated with AMS after adjusting for multiple additional potential confounders as follows: model 0, unadjusted; model 1, age, BMI, smoking, and drinking; and model 2, those in model 1 plus HR, MAP, SpO_2_, and E/A. The highest tertile of SVi was defined as the reference category. Statistical power calculations were performed by using the PASS software, version 11 (NCSS, LLC, Kaysville, UT, USA), suggesting that 238 AMS cases would provide more than 80% power to detect an IRR of at least 1.8 among SVi tertiles. All statistical tests were two-sided, and a *p* value of <0.05 was statistically significant. SPSS 17.0 (SPSS Inc., Chicago, IL, USA) was used to perform the statistical analysis.

## 3. Results

### 3.1. Differences of Basic Characteristics, Physiological Parameters, LV Function, and Mitral Doppler Flow in Subjects at Sea Level or at High Altitude with or without AMS

**Figure 2:**
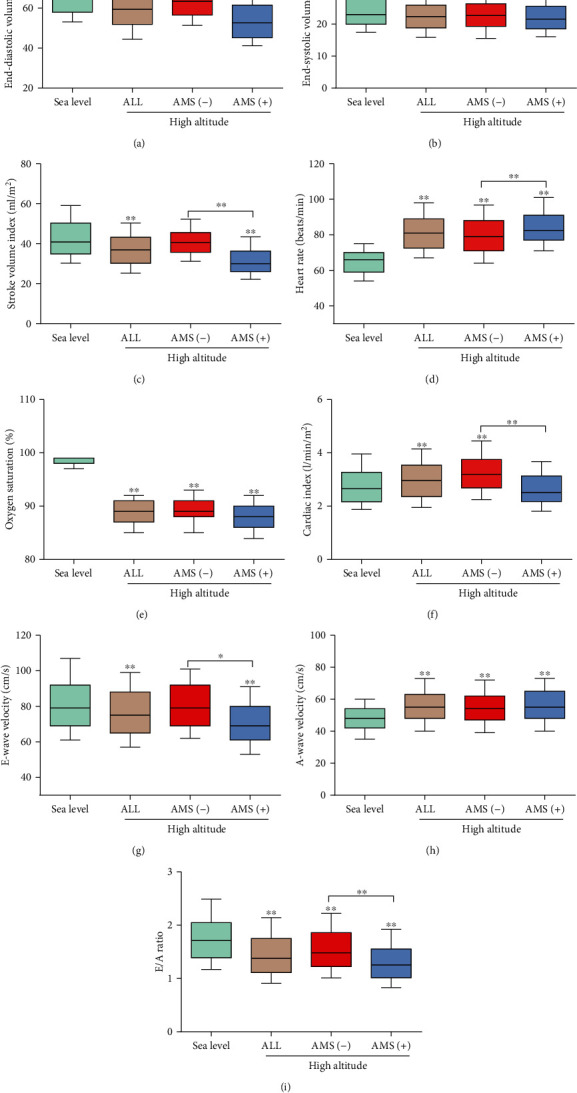
Changes of oxygen saturation and the main cardiac parameters in subjects at sea level or at high altitude with or without AMS: (a) EDVi; (b) ESVi; (c) SVI; (d) HR; (e) SpO_2_; (f) CI; (g) E-wave velocity; (h) A-wave velocity; (i) E/A ratio. Dark horizontal lines indicated median values, and the top and bottom of the boxes represent the 75th and 25th percentiles, respectively. The top and bottom whiskers represented the 90th and 10th percentiles, respectively. ALL: all the subjects at HA; AMS (-): subjects with non-AMS; AMS (+): AMS patients; other abbreviations are as in Table 1. ^∗^*p* < 0.05 and ^∗∗^*p* < 0.01 compared to the sea level groups or indicated groups.

The baseline information of 559 participants was available, whereas data from 30 participants were missing (5.1%). Among the 589 participants, 238 subjects (40.4%) were diagnosed with AMS, and the other 351 participants were not (Figure 1). As shown in Table 1, no significant differences in height, weight, BMI, BSA, and status of smoking and drinking had been found among all the groups. Nevertheless, AMS patients were older than AMS-free subjects. Moreover, following acute HA exposure, HR, DBP, and MAP were increased and SpO_2_ was decreased in all the groups; SBP was elevated in all subjects and AMS-free subjects but not in AMS patients; the ESVi, EDVi, and SVi were decreased in all subjects and AMS patients but not AMS-free subjects; LVEF was increased in all the groups, whereas CI was elevated in all subjects and AMS-free subjects but not in AMS patients; mitral peak E-wave velocity was decreased in all subjects and AMS patients but not in AMS-free subjects, whereas mitral peak A-wave velocity was increased in all the groups, which resulted in a significant reduction of the E/A ratio in all the groups. Interestingly, compared to AMS-free subjects, AMS patients showed higher HR and lower EDVi, SVi, E-wave velocity, and E/A ratio as well as insufficient CI (Figure 2).

### 3.2. The Discrimination Ability of Potential Variables for AMS Incidence

**Figure 3:**
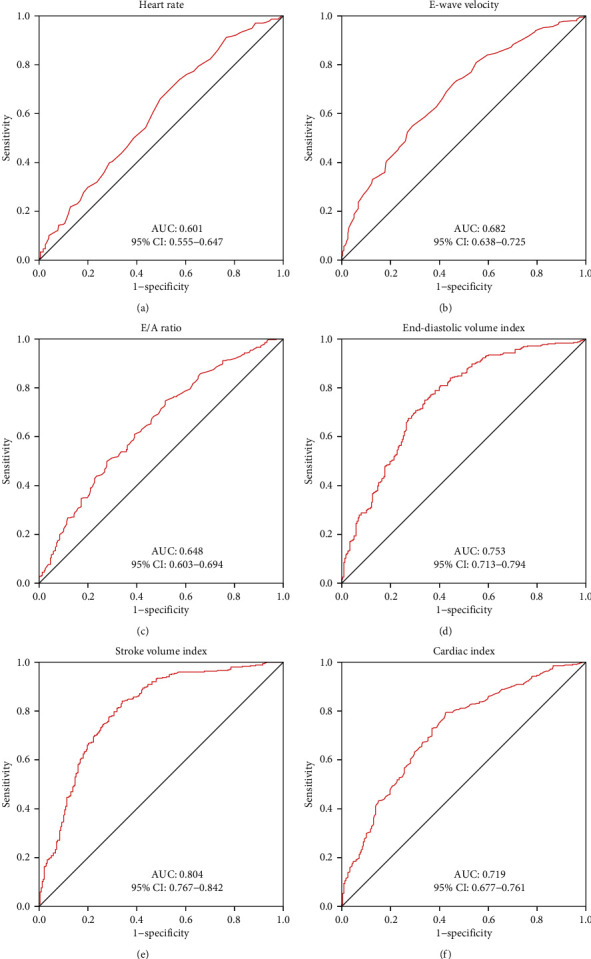
Receiver operator characteristic curves of HR (a), E-wave velocity (b), E/A ratio (c), EDVi (d), SVi (e), and CI (f) in discriminating AMS. AUC = area under the curve; CI = confidence interval; other abbreviations are as in Table 1.

ROC curve analysis was employed to further evaluate the discrimination ability of the above variables, which were different between AMS patients and AMS-free subjects. The area under the curve (AUC) was highest for SVi (AUC: 0.804, 95% CI: 0.767-0.842) compared with HR (AUC: 0.601, 95% CI: 0.555-0.647), E-wave velocity (AUC: 0.682, 95% CI: 0.638-0.725), E/A ratio (AUC: 0.648, 95% CI: 0.603-0.694), EDVi (AUC: 0.753, 95% CI: 0.713-0.794), and CI (AUC: 0.719, 95% CI: 0.677-0.761) (Figure 3).

### 3.3. The Incidence of AMS, Population Characteristics, Physiological Parameters, LV Function, and Mitral Doppler Flow in Different Levels of SVi

**Figure 4:**
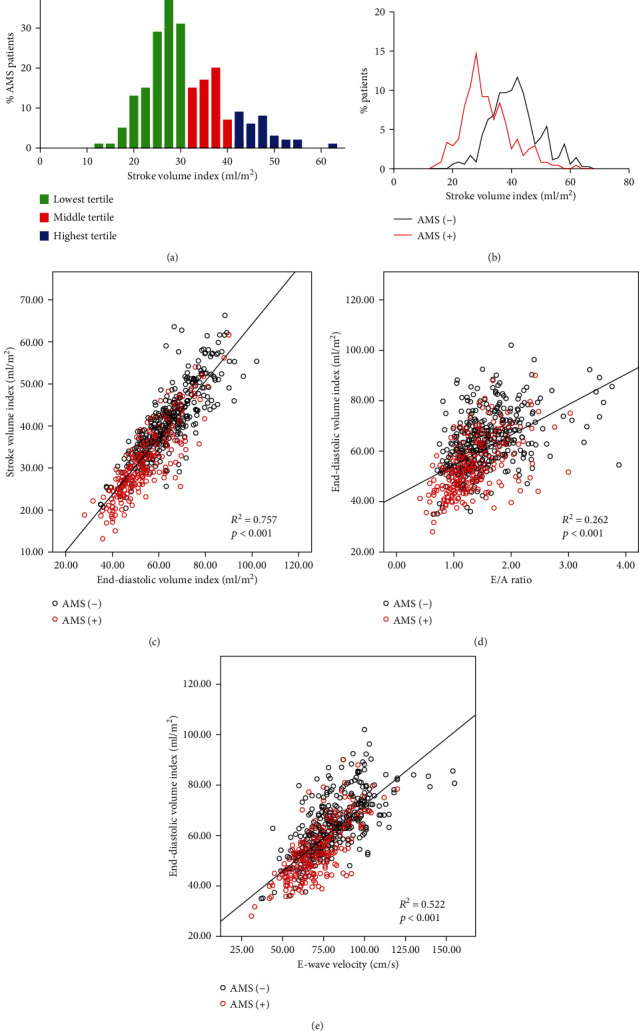
Distribution of stroke volume index (SVi) in subjects with or without acute mountain sickness (AMS) and its relationships with EDVi, E/A ratio, and E-wave velocity. (a) Incidence of AMS in different levels of SVi: green: lowest SVi tertile; red: middle SVi tertile; blue: highest SVi tertile. (b) The distribution of SVi in subjects with or without AMS. Linear regression analysis shows the correlations of SVi and EDVi (c), EDVi and E/A ratio (d), and EDVi and E-wave velocity (e).

A higher percentage of subjects in the lowest tertile of SVi developed AMS in comparison to subjects in the middle or highest tertile (Figure 4(a)). Moreover, the distribution of SVi in AMS patients showed a left shift in comparison to that observed in the non-AMS population (Figure 4(b)).

The basic characteristics, blood pressures, SpO_2_, HR, LVEF, and A-wave velocity of participants with SVi values in the highest and middle tertiles did not differ from each other. Compared with subjects in the highest SVi tertile, subjects in the lowest SVi tertile showed higher age and HR. With the decrease of SVi, the ESVi, EDVi, CI, E-wave velocity, and E/A ratio in the middle SVi tertile were concomitantly decreased and further declined in the lowest SVi tertile except for ESVi (Table 2).

Results from Pearson correlation analysis showed that SVi was negatively correlated with HR (*r* = −0.12, *p* < 0.01) and positively correlated with EDVi (*r* = 0.87, *p* < 0.01), E-wave velocity (*r* = 0.66, *p* < 0.01), and E/A ratio (*r* = 0.45, *p* < 0.01) (Supplemental Table 1). Linear regression analysis identified that the changes of SVi were mainly associated with the changes of EDVi (*R*^2^ = 0.757, *p* < 0.01) (Figure 4(c)), whereas the reductions of EDVi were significantly associated with the alterations of E/A ratios (*R*^2^ = 0.262, *p* < 0.01) (Figure 4(d)), which was mainly correlated with the changes of E-wave velocity (*R*^2^ = 0.522, *p* < 0.01) (Figure 4(e)).

### 3.4. The IRR of Continuous and Categorical SVi for AMS Incidence

When treating SVi as a continuous variable, the IRR values for AMS incidence were 0.932 (95% CI: 0.922-0.942), 0.933 (95% CI: 0.922-0.943), and 0.936 (95% CI: 0.925-0.948) in the unadjusted model, model 1, and model 2, respectively. When treating SVi as a categorical variable, in the unadjusted model, subjects in the middle SVi tertile (IRR: 1.958, 95% CI: 1.333-2.876) and the lowest SVi tertile (IRR: 4.710, 95% CI: 3.375-6.572) were more prone to develop AMS than subjects in the highest SVi tertile. Further analysis revealed that compared with subjects with the highest SVi tertile, the IRR for AMS incidence in subjects in the middle SVi tertile and the lowest SVi tertile were 1.964 (95% CI: 1.339-2.879) and 4.619 (95% CI: 3.311-6.444) by adjustment for model 1 and 1.872 (95% CI: 1.276-2.747) and 4.097 (95% CI: 2.892-5.803) by adjustment for model 2, respectively (Table 3).

### 3.5. The Clinical Severity of AMS Symptoms in Subjects with Different Levels of SVi

Consistently, compared to subjects in the highest SVi tertile, subjects with lower SVi attained a higher AMS score. Furthermore, subjects in the middle SVi tertile showed higher incidences of mild headache and gastrointestinal symptoms but not of other symptoms than did subjects in the highest SVi tertile. In addition, higher incidences of all symptoms except for moderate/severe gastrointestinal symptoms were observed in subjects in the lowest SVi tertile compared with subjects in the highest SVi tertile, whereas subjects in the lowest SVi tertile showed higher incidences of all the symptoms compared with subjects in the middle SVi tertile (Table 4).

## 4. Discussion

To date, this is the largest echocardiography-based case-control study of subjects with or without AMS following acute HA exposure. Our results demonstrated that the levels of SpO_2_, ESVi, EDVi, SVi, E-wave velocity, and E/A ratio were decreased, whereas the levels of HR, blood pressures, LVEF, CI, and A-wave velocity were increased in subjects after acute HA exposure. However, HR was higher, whereas the EDVi, SVi, CI, E-wave velocity, and E/A ratio were lower in AMS patients than that in AMS-free subjects. ROC analysis identified that SVi was the most valuable factor for the discrimination of AMS. Moreover, the change of SVi was mainly correlated with EDVi and altered LV filling pattern. Compared with subjects in the highest SVi tertile, subjects in the middle SVi tertile showed higher multivariable IRR for AMS with higher incidences of mild headache and gastrointestinal symptoms, whereas subjects in the lowest SVi tertile showed even higher multivariable IRR with higher incidences of all the symptoms. These results revealed that the reduction of SVi correlated with the altered LV filling pattern was associated with the incidence and clinical severity of AMS.

Acute HA exposure led to major alterations in cardiovascular function. Adaptive cardiac responses to acute HA hypoxia played pivotal roles in O_2_ and nutrient delivery in lowlanders during their sojourn to HA at rest or during exercise. Our previous study had shown that the initial cardiac responses included an increase in CO secondary to an elevation of HR so as to compensate for the reduced arterial oxygen content before acclimatization. After acclimatization for a few days, CO began to fall, but HR further increased, whereas SV decreased [16]. However, the characteristics of LV functional responses in AMS patients had not been illustrated so far. Our present results showed that the HR was higher but the EDVi, SVi, CI, E-wave velocity, and E/A ratio were lower in AMS patients than that in AMS-free subjects. These distinctive changes indicated that there might be some associations between these factors and AMS incidence.

ROC analysis identified that SVi was the most valuable factor for the discrimination of AMS. A previous study indicated that the fall of SVi was the comprehensive outcome of multiple factors including decreased blood volume, the increase of HR, and altered LV filling [17]. Moreover, our results showed that the HR of subjects in the lowest SVi tertile was the highest, but the CI was the lowest, which suggested that the alteration of SVi was of predominance. Additionally, due to the offset by HR, the AUC of CI for the discrimination of AMS was lower than that of SVi. Other factors including the altitude attained, degree of preacclimatization, rate of ascent, and individual susceptibility had also been proved to be associated with AMS incidence [11]. Moreover, subjects with migraine, mood states (such as anxiety), or obesity were also associated with the incidence of AMS, whereas subjects older than 50 years or women were more susceptible to AMS [18, 19]. However, most of these factors were inherent or unmodifiable. In this study, we identified that SVi as a protective factor was associated with AMS incidence in both the unadjusted and adjusted models, which provided new insight into the underlying mechanisms in AMS incidence and implied a novel strategy for the prevention and treatments for AMS.

The fall of SVi in AMS patients was mainly attributed to the reduction in EDVi (75.7% contribution) secondary to the decreased E-wave velocity and E/A ratio. These results suggested that the diastolic function was limited with the characteristic of altered LV filling pattern, which may partly be a consequence of diuresis and reduction of plasma volume, for the reason that the plasma volume decreased in the first weeks by nearly 20% at 3800 to 4500 m [4]. On the other hand, acute HA hypoxia-induced hypoxic pulmonary vasoconstriction (HPV) may also contribute to the fall of SVi [20], for the reason that the volume of blood returning to LV declined during each diastolic period. Consistently, our previous results showed that acute HA exposure induced borderline pulmonary hypertension, which suggested a transient HPV. However, the HA-induced borderline pulmonary hypertension showed no difference between subjects with AMS patients and AMS-free subjects [21]. These results suggested that the acute HA hypoxia-induced HPV might partly, but not mainly, account for the fall of SVi in AMS patients in the present study.

The dependence of myocardial relaxation on an adequate oxygen supply was a putative mechanism for the diastolic dysfunction in response to acute HA hypoxia [22]. A previous study demonstrated that an adult Sherpa who underwent a prolonged HA hypoxia exposure showed lower early transmitral filling, slower LV diastolic relaxation, and ultimately a smaller EDV compared with sea level inhabitants [23]. Moreover, acute hypoxia exposure increased the isovolumic relaxation time and decreased the mitral and tricuspid inflow and annuli E/A ratio [24], which suggested that the diastolic function was limited under both acute and chronic HA exposure. Thus, the limited LV diastolic function in AMS patients might be a consequence of impaired myocardial relaxation with a rapid decrease in high-energy phosphate metabolism during acute HA hypoxia [25]. Moreover, the increased left ventricular twist and torsion-to-shortening ratios which were associated with significant changes in LV dimensions and contractility following acute HA exposure suggested the occurrence of subendocardial systolic dysfunction albeit global LV function was normal [26]. This might also be a contributor to the altered LV filling pattern and the fall of SVi. Another study also showed that acute HA exposure delayed LV untwist, impaired LV diastolic function, and eventually decreased the LV filling, which was concomitant with the occurrence of AMS [27], suggesting a potential relationship between the limited LV diastolic function and AMS. In the present study, the fall of SVi in AMS patients might be attributed to the limited LV diastolic function with an altered LV filling pattern. Accordingly, patients with a variety of cardiovascular conditions including diastolic dysfunction should comply with the newly published recommendations [28] to avoid the occurrence of secondary cardiovascular events as well as circumstances conducive to AMS. Clinically, strategies for ameliorating diastolic dysfunction and enhancing SVi at HA might be of benefit for the prevention and treatment of AMS. Our results suggested a tendency that subjects with lower SVi are more likely to suffer AMS after acute high-altitude exposure. It may be helpful in the prevention of AMS. For subjects with typical symptoms but have not suffered AMS, if their levels of SVi are relatively lower, more attention should be addressed and appropriate treatment such as rest, oxygen supplementation, or acetazolamide should be taken to prevent the progress towards AMS. Furthermore, we can take measures to enhance SVi, thereby reducing the risk of AMS. Nevertheless, our conclusions still need to be verified by more researches.

The strengths of the present study include a larger sample size than ever before and a solid, standardized methodology. However, the observed results should be explained with some limitations in mind. First, the enrolled participants were young healthy men; whether the established results could extend to other types of individuals or circumstances (such as women, older adults, children, and ascent to HA by car or other transport modes) is still unknown. Second, the classification of AMS was based on a self-report without immediate medical control, which might lead to possible classification bias. Last, 2-dimensional echocardiography, like other imaging tools, has inherent limitations impeding a good evaluation of complex 3-dimensional anatomy. Nevertheless, echocardiography remains the simple and feasible method for field studies at HA. In the present study, the same well-trained operators who adopted strict reading criteria and were blind to the subjects' grouping information performed all examinations. Therefore, such an approach could reduce the above-mentioned limitations to the utmost.

## 5. Conclusions

This relatively large-scale case-control study demonstrated that AMS patients showed lower SVi than AMS-free subjects. The reduction of SVi in AMS patients correlated with the altered LV filling pattern was significantly associated with the incidence and clinical severity of AMS. These results provide novel insights into the underlying mechanisms in the occurrence of AMS and new strategies for the prevention and treatment of AMS.

## Figures and Tables

**Table 1:** Basic characteristics, physiological parameters, left ventricular function, and mitral Doppler flow in subjects at sea level or at HA with or without AMS.

Variables	Sea level (*n* = 559)	High altitude (*n* = 589)		
		ALL	AMS (-)	AMS (+)
		(*n* = 589)	(*n* = 351)	(*n* = 238)
*Basic characteristics*				
Age (years)	23 (20-26)	23 (20-26)	22 (20-24)	23 (20-27)^##^
Height (cm)	172 (168-175)	172 (168-175)	172 (169-175)	172 (168-175)
Weight (kg)	64 (59-69)	64 (59-69)	64 (60-68)	64 (58-70)
BMI (kg·cm^−2^)	21.6 (20.2-23.2)	21.5 (20.2-23.1)	21.6 (20.2-23.1)	21.5 (20.1-23.2)
BSA (m^2^)	1.71 (1.63-1.79)	1.71 (1.63-1.79)	1.71 (1.64-1.78)	1.71 (1.62-1.80)
Smoking (*N*, %)	378 (67.6%)	403 (68.4%)	240 (68.4%)	163 (68.5%)
Drinking (*N*, %)	353 (63.1%)	371 (63.0%)	225 (64.1%)	146 (61.3%)
*Physiological parameters*				
HR (bpm)	66 (59-70)	81 (73-89)^∗∗^	79 (71-88)^∗∗^	83 (77-91)^∗∗^^##^
SBP (mmHg)	115 (108-122)	118 (111-124)^∗^	118 (111-123)^∗^	117 (109-124)
DBP (mmHg)	74 (68-80)	78 (72-85)^∗∗^	78 (72-85)^∗∗^	78 (72-84)^∗∗^
MAP (mmHg)	87 (82-94)	91 (85-97)^∗∗^	91 (86-98)^∗∗^	91 (84-96)^∗∗^
SpO_2_ (%)	98.0 (98.0-99.0)	89.0 (87.0-91.0)^∗∗^	89.0 (88.0-91.0)^∗∗^	88.0 (86.0-90.0)^∗∗^
*LV function*				
ESVi (ml/m^2^)	23.0 (20.0-27.8)	22.4 (18.8-25.9)^∗∗^	22.7 (19.3-26.3)	21.6 (18.5-25.6)^∗∗^
EDVi (ml/m^2^)	65.6 (58.0-74.7)	59.4 (51.8-68.2)^∗∗^	63.4 (56.5-71.5)	52.6 (45.2-61.5)^∗∗^^##^
SVi (ml/m^2^)	40.9 (34.9-50.3)	37.0 (30.2-43.3)^∗∗^	40.7 (35.7-45.6)	30.0 (26.0-36.4)^∗∗^^##^
LVEF (%)	63.7 (62.4-66.5)	66.6 (64.0-70.0)^∗∗^	66.7 (64.0-70.0)^∗∗^	66.5 (64.0-69.0)^∗∗^
CI (l/min/m^2^)	2.7 (2.2-3.3)	3.0 (2.4-3.5)^∗∗^	3.2 (2.7-3.8)^∗∗^	2.5 (2.2-3.1)^##^
*Mitral Doppler flow*				
E-wave velocity (cm/s)	79 (69-92)	75 (65-88)^∗∗^	79 (69-92)	69 (61-80)^∗∗^^##^
A-wave velocity (cm/s)	48 (42-54)	55 (48-63)^∗∗^	54 (47-62)^∗∗^	55 (48-65)^∗∗^
E/A ratio	1.71 (1.39-2.05)	1.38 (1.11-1.75)^∗∗^	1.48 (1.22-1.86)^∗∗^	1.23 (1.02-1.55)^∗∗^^##^

Values are expressed as the median (interquartile range) or *n* (%). AMS: acute mountain sickness; BMI: body mass index; BSA: body surface area; HR: heart rate; SBP: systolic blood pressure; DBP: diastolic blood pressure; MAP: mean arterial pressure; SpO_2_: oxygen saturation; ESVi: end-systolic volume index; EDVi: end-diastolic volume index; SVi: stroke volume index; LVEF: left ventricular ejection fraction; CI: cardiac index; E: peak flow velocity of the early mitral filling wave; A: peak flow velocity of late mitral filling wave; bpm: beats per minute. ^∗^*p* < 0.05, ^∗∗^*p* < 0.01 vs. subjects at sea level and ^#^*p* < 0.05 and ^##^*p* < 0.01 vs. AMS (-) subjects.

**Table 2:** Basic characteristics, physiological parameters, left ventricular size, and function in subjects in different tertiles of SVi.

Variables	Highest tertile	Middle tertile	Lowest tertile
	(*n* = 196)	(*n* = 197)	(*n* = 196)
*Basic characteristics*			
Age (years)	22 (20-25)	22 (20-25)	23 (20-27)^∗^
Height (cm)	172 (168-175)	171 (169-174)	172 (168-175)
Weight (kg)	64 (59-68)	64 (59-68)	64 (59-70)
BMI (kg·cm^−2^)	21.4 (20.0-23.1)	21.8 (20.3-23.2)	21.5 (20.2-23.2)
BSA (m^2^)	1.71 (1.64-1.78)	1.71 (1.63-1.78)	1.71 (1.63-1.79)
Smoking (*N*, %)	138 (70.4%)	132 (67.0%)	133 (67.9%)
Drinking (*N*, %)	126 (64.3%)	128 (65.0%)	117 (60.0%)
*Physiological parameters*			
HR (bpm)	80 ± 13	81 ± 12	84 ± 13^∗∗^^#^
SBP (mmHg)	118 (111-123)	118 (111-124)	117 (111-123)
DBP (mmHg)	78 (72-85)	78 (71-85)	78 (73-83)
MAP (mmHg)	91 (86-98)	91 (84-97)	91 (86-96)
SpO_2_ (%)	89.0 (87.3-91.0)	89.0 (87.0-91.0)	89.0 (87.0-91.0)
*LV function*			
ESVi (ml/m^2^)	23.6 (20.7-27.2)	22.0 (18.4-25.9)^∗∗^	21.0 (18.1-24.5)^∗∗^
EDVi (ml/m^2^)	72.2 ± 8.8	59.5 ± 6.5^∗∗^	48.7 ± 7.6^∗∗^^##^
SVi (ml/m^2^)	46.0 (43.3-51.3)	37.0 (35.2-39.0)^∗∗^	27.7 (24.4-30.2)^∗∗^^##^
LVEF (%)	66.0 (64.0-70.0)	67.0 (64.1-70.3)	66.1 (64.0-69.0)
CI (l/min/m^2^)	3.83 ± 0.75	3.00 ± 0.50^∗∗^	2.25 ± 0.45^∗∗^^##^
*Mitral Doppler flow*			
E-wave velocity (cm/s)	89.5 (78.0-99.0)	75.0 (68.0-83.5)^∗∗^	64.0 (58.0-72.8)^∗∗^^##^
A-wave velocity (cm/s)	53.0 (46.3-60.8)	54.0 (47.0-63.0)	56.0 (48.3-65.0)
E/A ratio	1.67 (1.37-1.98)	1.40 (1.14-1.74)^∗∗^	1.17 (0.96-1.39)^∗∗^^##^

Values are expressed as the mean ± SD or the median (interquartile range); abbreviations are the same as in Table 1. ^∗^*p* < 0.05 and ^∗∗^*p* < 0.01 vs. subjects in the highest SVi tertile. ^#^*p* < 0.05 and ^##^*p* < 0.01 vs. subjects in the middle SVi tertile.

**Table 3:** The incidence of AMS according to SVi.

	Incidence of AMS, IRR (95% CI)			
		Decreasing category of SVi		
	Continuous	Highest tertile	Middle tertile	Lowest tertile
No. of cases	238	31	61	146
No. of controls	351	165	136	50
Model 0	0.932 (0.922-0.942)	1 (reference)	1.958 (1.333-2.876)	4.710 (3.375-6.572)
Model 1	0.933 (0.922-0.943)	1 (reference)	1.964 (1.339-2.879)	4.619 (3.311-6.444)
Model 2	0.936 (0.925-0.948)	1 (reference)	1.872 (1.276-2.747)	4.097 (2.892-5.803)

IRR = Incidence Rate Ratios; CI = confidence interval; other abbreviations are as in Table 1. Model 0: unadjusted; model 1: adjusted by age, BMI, smoking, and drinking; model 2: adjusted by model 1 plus HR, MAP, SpO_2_, and E/A.

**Table 4:** The AMS scores and clinical symptoms of subjects with different tertiles of SVi.

	Highest tertile	Middle tertile	Lowest tertile
	(*n* = 196)	(*n* = 197)	(*n* = 196)
AMS score	2.0 (0.3-2.0)	2.0 (1.0-3.0)^∗^	3.0 (3.0-4.0)^∗∗^^##^
*Headache*			
Mild	69 (35.2%)	90 (45.7%)^∗^	125 (63.8%)^∗∗^^##^
Moderate/severe	9 (4.6%)	12 (6.1%)	41 (20.9%)^∗∗^^##^
*Dizziness*			
Mild	82 (41.8%)	98 (49.7%)	126 (64.3%)^∗∗^^#^
Moderate/severe	8 (4.1%)	11 (5.6%)	30 (15.3%)^∗∗^^##^
*Gastrointestinal symptoms*			
Mild	22 (11.2%)	37 (18.8%)^∗^	60 (30.6%)^∗∗^^##^
Moderate/severe	2 (1.0%)	0 (0.0%)	6 (3.1%)^#^
*Fatigue*			
Mild	94 (48.0%)	94 (47.7%)	122 (62.2%)^∗∗^^##^
Moderate/severe	9 (4.6%)	10 (5.1%)	35 (17.9%)^∗∗^^##^

Values are expressed as the median (interquartile range) or *n* (%), ^∗^*p* < 0.05 and ^∗∗^*p* < 0.01 vs. subjects in the highest SVi tertile. ^#^*p* < 0.05 and ^##^*p* < 0.01 vs. subjects with subjects in the middle SVi tertile.

## Data Availability

The data used to support the findings of this study are available from the corresponding author upon request.

## Supplementary Materials

Supplementary Materials Supplemental Table 1: pairwise Pearson correlation coefficients between the factors associated with AMS incidence.
