# Supplementary material for: Low Stroke Volume Index in Healthy Young Men Is Associated with the Incidence of Acute Mountain Sickness after an Ascent by Airplane: A Case-Control Study
Source: Biomed Res Int. 2020 Nov 10;2020:6028747. doi: 10.1155/2020/6028747 (PMC7673943; doi:10.1155/2020/6028747)
Supplement: Supplementary Materials — Supplemental Table 1: pairwise Pearson correlation coefficients between the factors associated with AMS incidence. [file 6028747.f1.docx]

**Supplemental Table 1. Pairwise Pearson correlation coefficients between the factors associated with AMS incidence.**

|  | HR | SpO_2_ | MAP | EDVi | ESVi | SVi | E | A | E/A |
| --- | --- | --- | --- | --- | --- | --- | --- | --- | --- |
| HR | 1.00 |  |  |  |  |  |  |  |  |
| SpO_2_ | -0.21** | 1.00 |  |  |  |  |  |  |  |
| MAP | 0.15** | -0.01 | 1.00 |  |  |  |  |  |  |
| EDVi | -0.14** | 0.02 | 0.03 | 1.00 |  |  |  |  |  |
| ESVi | -0.10* | 0.00 | 0.04 | 0.65** | 1.00 |  |  |  |  |
| SVi | -0.12** | 0.03 | 0.00 | 0.87** | 0.19** | 1.00 |  |  |  |
| E | -0.10* | -0.01 | -0.01 | 0.72** | 0.43** | 0.66** | 1.00 |  |  |
| A | 0.21** | -0.10* | 0.16** | -0.10* | -0.09* | -0.07 | -0.06 | 1.00 |  |
| E/A | -0.19** | 0.05 | -0.13** | 0.51** | 0.33** | 0.45** | 0.66** | -0.71** | 1.00 |

* p<0.05, **p<0.01, abbreviations were the same in Table 1.
